# A Comparative Survey of Anti-Melanoma and Anti-Inflammatory Potential of Usnic Acid Enantiomers—A Comprehensive In Vitro Approach

**DOI:** 10.3390/ph14090945

**Published:** 2021-09-21

**Authors:** Agnieszka Galanty, Paweł Zagrodzki, Joanna Gdula-Argasińska, Karolina Grabowska, Paulina Koczurkiewicz-Adamczyk, Dagmara Wróbel-Biedrawa, Irma Podolak, Elżbieta Pękala, Paweł Paśko

**Affiliations:** 1 Department of Pharmacognosy, Faculty of Pharmacy, Jagiellonian University Medical College, Medyczna 9, 30-688 Kraków, Poland; karolina1.grabowska@uj.edu.pl (K.G.); dagmara.wrobel-biedrawa@uj.edu.pl (D.W.-B.); irma.podolak@uj.edu.pl (I.P.); 2 Department of Food Chemistry and Nutrition, Faculty of Pharmacy, Jagiellonian University Medical College, Medyczna 9, 30-688 Kraków, Poland; pawel.zagrodzki@uj.edu.pl (P.Z.); p.pasko@uj.edu.pl (P.P.); 3 Department of Radioligands, Faculty of Pharmacy, Jagiellonian University Medical College, Medyczna 9, 30-688 Krakow, Poland; joanna.gdula-argasinska@uj.edu.pl; 4 Department of Pharmaceutical Biochemistry, Faculty of Pharmacy, Jagiellonian University Medical College, Medyczna 9, 30-688 Kraków, Poland; paulina.koczurkiewicz@uj.edu.pl (P.K.-A.); elzbieta.pekala@uj.edu.pl (E.P.)

**Keywords:** usnic acid enantiomers, melanoma, cytotoxic, isobolographic analysis, anti-inflammatory, tyrosinase, hyaluronidase

## Abstract

Usnic acid (UA) is a chiral lichen metabolite with an interesting pharmacological profile. The aim of this study was to compare the anti-melanoma effect of (+)-UA and (−)-UA in an in vitro model by studying their impact on the cells as well as the processes associated with cancer progression. The effect of UA enantiomers on the viability, proliferation, and invasive potential of three melanoma cell lines (HTB140, A375, WM793) was evaluated. Their interaction with a chemotherapeutic drug—doxorubicin was assessed by isobolographic analysis. Anti-inflammatory and anti-tyrosinase properties of (+)-UA and (−)-UA were also examined. Both UA enantiomers dose- and time-dependently decreased the viability of all three melanoma cell lines. Their synergistic effect with doxorubicin was observed on A375 cells. (+)-Usnic acid at a sub-cytotoxic dose strongly inhibited melanoma cells migration. Both UA enantiomers decreased the release of pro-inflammatory mediators. The cytotoxic effect of (+)-UA and (−)-UA depends greatly on the melanoma cell type; however, the overall anti-melanoma potential is perspective. Our results indicate that the strategy of combining usnic acid enantiomers with cytostatic drugs may be an interesting option to consider in combating melanoma; however, further studies are required.

## 1. Introduction

Melanoma is a form of skin cancer characterised by rapid development, poor prognosis, and high mortality. It is the most dangerous type of skin cancer due to its prominent metastasis and resistance to radio- and chemotherapy [1,2]. Although much effort has been made worldwide, the effectiveness of anti-melanoma therapy is still not satisfactory. One of the reasons for chemotherapy resistance and treatment failures is the phenotypic heterogeneity of tumour cells. Hanahan and Weinberg (2011) suggested that cancer cells within one tumour, despite some functional similarities, can adopt different strategies for promotion and progression, resulting in not uniform proliferation rate and, in consequence, a varied response of the cells to chemotherapeutics [3]. Other symptoms, like inflammation, concomitant with cancer, may also affect therapy effectiveness. Moreover, in the case of melanoma, the expression of tyrosinase, a key enzyme in melanin synthesis, plays an important role in the progression of the disease as it may decrease cellular response to radiotherapy [4]. Thus, the preselection of new candidates for anti-cancer agents should be focused not only on the mechanisms affecting different cellular functions crucial for the development of cancer but also on reducing the co-existing symptoms [5]. Such a comprehensive approach has a greater chance to develop more effective cancer treatment options.

Usnic acid (2,6-diacetyl-7,9-dihydroxy-8,9b-dimethyl-1,3(2*H*,9b*H*)-dibenzo-furandione) is one of the most widely studied lichen metabolites, with a wide array of pharmacological activities, of which cytotoxic, anti-inflammatory and analgesic, are especially well documented [6]. Therefore, usnic acid has been particularly widely studied for its anti-cancer potential, and the results seem to be very promising, as it shows a marked activity towards different cancers, both in vitro and in vivo [7]. Usnic acid is a chiral compound, and its two enantiomers can be found in natural sources in varied ratios. The question regarding potential differences in the activity of (+)- and (−)-usnic acid is still open, as the evidence provided in literature reports is scarce and ambiguous [8]. This is an important issue as the effectiveness and toxicity of many chiral compounds may vary significantly. Even though a large body of data refers to the anti-cancer effects of usnic acid, its anti-melanoma activity has been reported so far in only a few studies [9,10,11,12], all of which are concerned solely with the right-handed enantiomer. On the other hand, hepatotoxic properties of both usnic acid enantiomers were described [7,13], which may limit their internal administration. It seems, therefore, that topical application of usnic acid could be a better option for its future use. In our previous work, we proved the high safety of both enantiomers to different normal skin cells and also indicated their good permeability through the skin barrier [14], which is essential for effectiveness when used topically. These results prompted us to widen our research and to examine the potential of both usnic acid enantiomers in skin cancer. Thus, the aim of the current study was to compare the anti-melanoma effect of (+)- and (−)-usnic acid in an in vitro model by studying, not only their direct impact on the cells, but also the influence on the processes associated with cancer progression. To achieve this, we examined the effect of both enantiomers on the viability, proliferation and invasive potential of three melanoma cell lines (HTB140, A375, WM793), differing in origin and metastatic potential, to mimic the complex phenotypic heterogeneity of the tumour itself. Moreover, isobolographic analysis was employed to study the interaction of (+)- and (−)-usnic acid with a chemotherapeutic drug—doxorubicin. Finally, to complete the research bearing in mind a comprehensive approach to anti-melanoma therapy, the anti-inflammatory and anti-tyrosinase properties of usnic acid enantiomers were also examined. 

## 2. Results and Discussion 

### 2.1. Both Usnic Acid Enantiomers Dose- and Time-Dependently Decreased Melanoma Cells Viability 

In the first step of the experiment, the impact of usnic acid enantiomers on the viability of HTB140, A375 and WM793 melanoma cells was determined, not only after standard 24 h but also after prolonged 48 h exposure (Table 1). Both compounds significantly affected the viability of the tested cell lines in a dose- and time-dependent manner, but in general (+)-usnic acid revealed a stronger cytotoxic effect (*p* < 0.001) than its left-handed counterpart. Among the cell lines tested, malignant melanoma A375 was most susceptible to (+)-usnic acid, with IC_50_ 11.84 µg/mL after 48 h, respectively, while for highly metastatic HTB140 cells, the observed effect was less pronounced (IC_50_ 14.72 µg/mL). What is interesting, both compounds affected the viability of primary melanoma WM793, which was not susceptible to doxorubicin (IC_50_ > 100 µg/mL); however, the effect was moderate (Table 1). 

To date, only a few reports concerned cytotoxic activity of usnic acid towards melanoma cells, but none of them compared the effectiveness of both enantiomers. Moreover, all the previously published reports were performed on other melanoma cell lines, and the obtained results were ambiguous. Brandao et al. (2012) described a weak cytotoxic effect of (+)-usnic acid to UACC-62 malignant melanoma cells (IC_50_ 184 µg/mL, 48 h). Other authors reported high activity of usnic acid against FemX (IC_50_ 12.72 µg/mL, 72 h), derived from lymph node as metastatic site, and 518A2 (IC_50_ 5.4 µM, about 2 µg/mL, 72 h), derived from unspecified metastatic site melanoma cells [11,12], however no information on the enantiomer used was provided. Our results, supported by the results of other authors, showed that the cytotoxic effect of both usnic acid enantiomers profoundly depends on the melanoma cell type, while the exerted effect seems to be promising. 

### 2.2. Both Usnic Acid Enantiomers Differently Interact with Doxorubicin Towards Melanoma Cells

**Figure 1:**
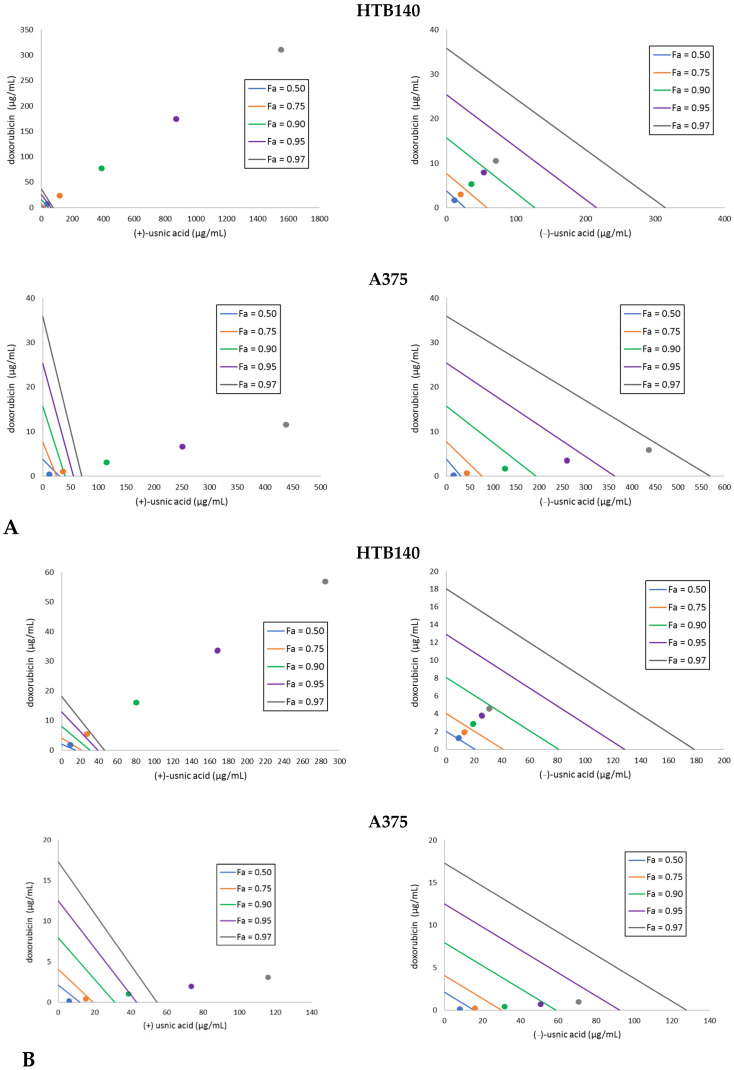
Isobolograms for the combinations of usnic acid enantiomers with doxorubicin to HTB140 and A375 melanoma cells, after 24 h (**A**) and 48 h (**B**) of incubation. Points on x- and y-axes, connected by the line, represent median doses (Fa = 0.50 means IC_50_, Fa = 0.75 means IC_75_, Fa = 0.90 means IC_90_, Fa = 0.95 means IC_95_, Fa = 0.97 means IC_97_) of the substances given alone, whereas the dots represent appropriate combinations of both substances, respectively. Dots below the lines of the same colour indicate synergy, dots on the line of the same colour indicate additive effect, whereas the dots over the lines of the same colour indicate antagonism of the interaction.

It is well known that a combination of two drugs may result in an increased therapeutic effect, which allows for lowering their doses and subsequently lead to fewer side effects. Thus, we decided to combine the tested usnic acid enantiomers with the reference cytostatic drug doxorubicin and determine the type of the interaction by means of isobolographic analysis. Doxorubicin was combined with either (+)- or (−)-usnic at a fixed ratio (1:1) of 1, 1/2, 1/4, 1/6, 1/8 of the respective IC_50_ values. The results are presented in Table 2 and Figure 1. A synergistic effect was obtained for the combination of both usnic acid enantiomers with doxorubicin against A375 cells. A predominance of the effect for the combination with (−)-usnic acid was seen, especially after 24 h of incubation (Figure 1). The most interesting observation was noted for WM793, which is the cell line not susceptible to doxorubicin. Here, a strong synergistic effect with (−)-usnic acid was noted at higher doses (CI 0.003–0.15) after 24 h, while for (+)-usnic acid, the observed synergism at higher doses (CI 0.75) finally changed into antagonism at lower doses (CI 1.37–1.85). After 48 h of incubation, the CI decreased, but the tendency remained the same (Table 2). 

For HTB140 cells, a strong antagonism was observed for (+)-usnic acid combined with doxorubicin, with the tendency to change into additive after longer incubation time, while the effect for (−)-usnic acid changed from synergistic (24 h) through additive and finally antagonistic (48 h). Thus, even though each cell line responded differently to the combination of usnic acid enantiomers with doxorubicin, our results indicate that the dose of the drug may be reduced. 

The strategy of combining usnic acid with a cytostatic drug has been rarely tackled [15,16,17,18], but the results are promising. The combination of usnic acid with sorafenib resulted in a synergistic effect on hepatocellular carcinoma cells, with CI 0.214 to 0.903. At higher usnic acid doses (100 µM), an antagonistic effect appeared (CI 1.26 to 2.33) [17]. A synergistic effect of (+)-usnic acid and tamoxifen in prostate cancer LNCaP was also noted (CI 0.14–0.73), while at higher doses of usnic acid (>500 µM), this combination had an antagonistic effect (CI 1.96 to 2.11) towards breast cancer MCF-7 cells [15,16]. The activity of paclitaxel to lung squamous carcinoma was enhanced by (+)-usnic acid, both in vitro and in vivo, but no CI was calculated [18]. What is important, all the cited experiments were performed only with (+)-usnic acid, after standard 24 h of incubation, thus the results obtained in the current study demonstrated for the first time an interesting effect also for the left-handed enantiomer, which has been less studied so far. Moreover, the role of longer incubation time was found to be significant. Our results clearly indicate that the strategy of combining usnic acid enantiomers with cytostatic drugs may also be an interesting option to consider in combating melanoma.

### 2.3. (+)-Usnic Acid Inhibited Proliferation of Melanoma Cells More Effectively Than (−)-Usnic Acid

**Figure 2:**
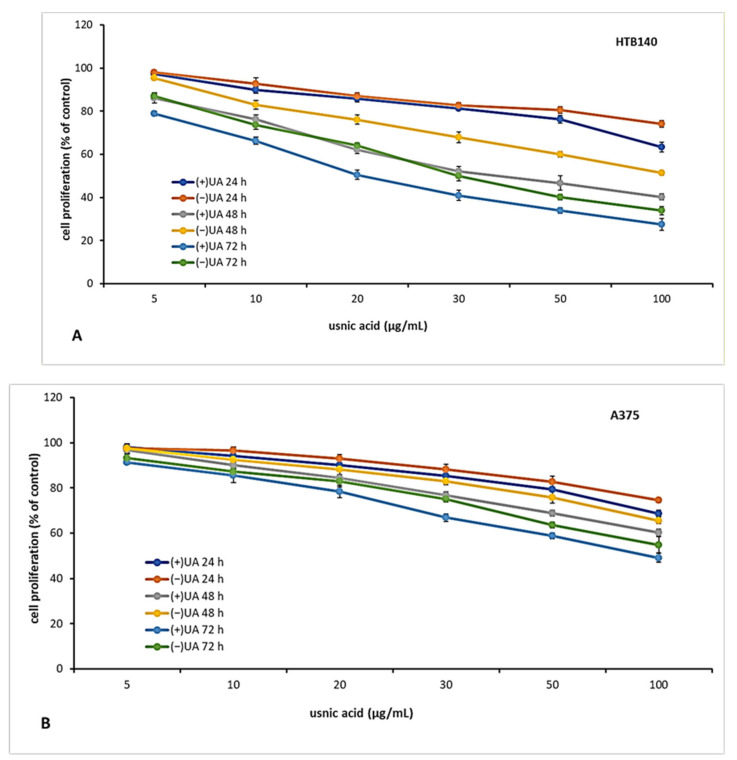

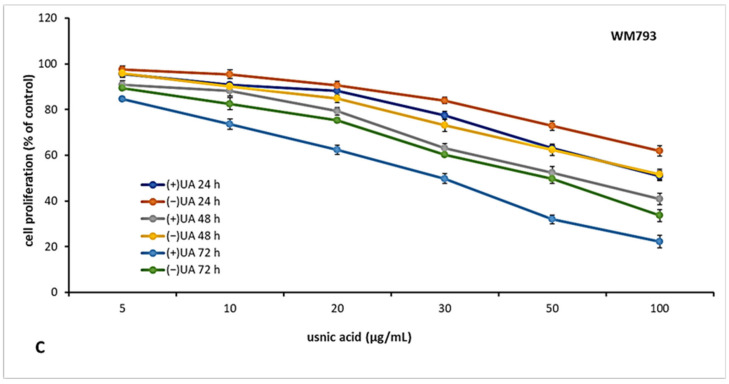
The effect of (+)- and (−)-usnic acid on proliferation of HTB140 (**A**), A375 (**B**) and WM793 (**C**) human melanoma cells.

The candidate for an anti-cancer drug should be characterised not only by cytotoxic but also cytostatic properties, which are essential to prevent tumour growth and development. Thus, in the next step of the experiment, we decided to verify the antiproliferative effect of usnic acid enantiomers towards the tested melanoma cell lines after 24, 48 and 72 h of incubation. The results are presented in Figure 2. Both usnic acid enantiomers revealed dose- and time-dependent cytostatic effect, with (+)-usnic acid being more effective. Similarly to cytotoxicity assay results, the response of the tested melanoma cell lines was varied. The highest inhibition of proliferation was noted for HTB140 cells (Figure 2A), and the effect was especially apparent after 72 h of incubation (IC_50_ 19.9 µg/mL and 31.1 µg/mL for (+)- and (−)-usnic acid, respectively). Also, the most significant differences between the activity of both enantiomers to HTB140 cells were observed after 48 h and 72 h of incubation, at concentrations above 20 µg/mL (*p* < 0.001). Antiproliferative activity of both enantiomers was also observed on WM793 cells (Figure 2C), with the most pronounced effect seen after 72 h (IC_50_ 28.6 µg/mL and 50.2 µg/mL for (+)- and (−)-usnic acid, respectively). Interestingly, A375 melanoma cells (Figure 2B), which were most vulnerable to cytotoxic action of both compounds, were, at the same time, the most resistant in terms of proliferation inhibition (IC_50_ > 100 µg/mL, for all tested conditions). Similar and even stronger cytostatic effect of (+)-and (−)-usnic acid was described for human breast T47-D (IC_50_ 4.2 µg/mL and 4.0 µg/mL, respectively) and pancreatic adenocarcinoma Capan-2 (IC_50_ 5.3 µg/mL and 5.0 µg/mL, respectively) cells [19], while no report exists on their influence on proliferation of any melanoma cells, thus our results may contribute to the development of a potential future anti-melanoma strategy. 

### 2.4. (+)-Usnic Acid at a Sub-Cytotoxic Dose Strongly Inhibited Melanoma Cells Migration

**Figure 3:**
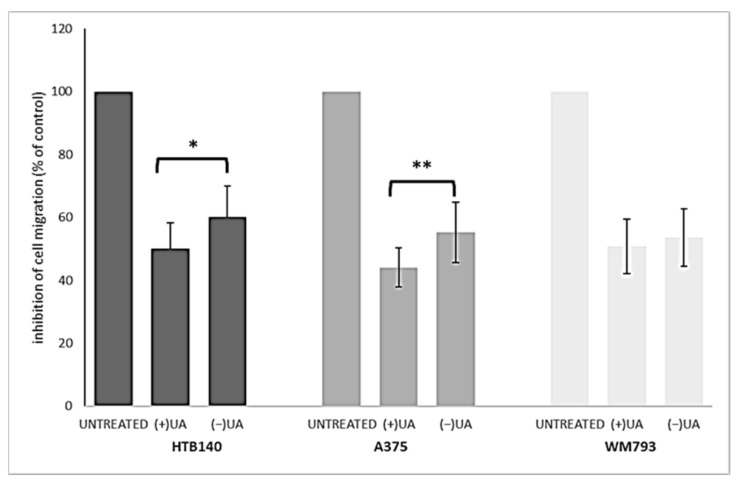
The effect of (+)- and (−)-usnic acid on migration ability of HTB140, A375 and WM793 human melanoma cells. Cells were treated or not (untreated) with 10 µg/mL of (+)-usnic acid (+UA) or (−)-usnic acid (−UA) for 24 h. Values are presented as the mean ± SD (standard deviation) of 10 randomly selected fields of view. Experiments were run twice in a blindfolded manner. Statistical analyses were carried out by using one-way ANOVA and Tukey post-hoc test. Significant differences (* *p* < 0.05 and ** *p* < 0.01) between usnic acid enantiomers were marked by upper black line.

Migration of cancer cells leads to their propagation from the primary site to other organs and is responsible for the further progress of a neoplastic disease. In order to better characterise usnic acid enantiomers as potential anti-melanoma agents, we performed a transwell migration assay for each of the tested melanoma cell lines. Based on the results of our previous work [9], we have chosen only one, sub-cytotoxic, dose of the tested compounds for the current experiment. Both usnic acid enantiomers strongly inhibited migration of the tested melanoma cells (Figure 3), at the dose as low as 10 µg/mL, when compared to the control, untreated cells (*p* < 0.001). 

Right-handed usnic acid was more potent than its left-handed enantiomer as migration inhibitor of HTB140 (*p* < 0.05) and A375 (*p* < 0.01) cells, while in the case of WM793 cells, the difference was not statistically significant. A strong suppression of cancer cells motile activity by (+)-usnic acid was described previously for human A549 lung, AGS gastric and HCT116 and LS174 colorectal cancer cell lines [20,21,22], while no data exists for any melanoma cells. Moreover, our results are the first to describe such activity for (−)-usnic acid. 

### 2.5. None of Usnic Acid Enantiomers Effectively Inhibited Tyrosinase Activity

Tyrosinase is a key enzyme controlling the synthesis of melanin, the overexpression of which may result in a decreased sensitivity of melanoma cells to therapy [4]. Thus, to verify yet another issue that may be of importance in the assessment of the anti-melanoma potential of usnic acid enantiomers, their inhibitory activity on tyrosinase activity was examined. We have chosen L-tyrosine and L-DOPA as substrates for the enzymatic assay to test the influence on both monophenolase and diphenolase activity of the enzyme. The obtained results show that usnic acid enantiomers have low activity, with a slightly higher effect observed for (+)-usnic acid. At the highest tested dose of 250 µg/mL, (+)-usnic acid revealed 5.22% and 9.1% tyrosinase inhibition, (−)-usnic acid 1.4% and 4.09%, while IC_50_ values for the control kojic acid were 1.1 µg/mL and 44.9 µg/mL, for mono- and diphenolase activity, respectively. Similar observations were recently described for (+)-usnic acid, with no anti-tyrosinase activity up to 50 [23] and 200 µM [24], while no information was available on the activity of its left-handed enantiomer. 

### 2.6. Both Usnic Acid Enantiomers Decreased Pro-Inflammatory Mediators Release

Inflammation of the skin tissue may increase the risk of melanoma. At the same time, the existing melanoma cells, by secreting a variety of pro-inflammatory factors, attract inflammatory cells, which enables the progression of metastasis and decreases the response to therapy [5,25,26]. It seems that a good candidate for an anti-melanoma drug, except for having a direct cellular effect, should also reveal anti-inflammatory properties, as cytokines involved in cancer-related inflammation are a promising target for a therapeutic approach. Thus, we decided to examine if usnic acid enantiomers, at sub-cytotoxic doses, may influence the release of selected pro-inflammatory mediators.

**Figure 4:**
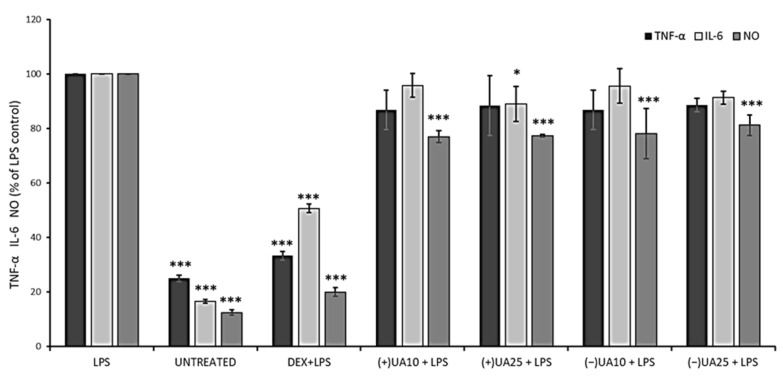
The effect of (+)- and (−)-usnic acid on tumour necrosis factor-alpha (TNF-α), interleukin-6 (IL-6) and nitric oxide (NO) release in LPS-stimulated RAW 264.7 macrophages. RAW cells were pre-treated with 10 (UA10) and 25 (UA25) µg/mL of usnic acid enantiomers for 1 h, afterwards cells were incubated with (10 ng/mL) or without LPS (untreated) for the next 24 h. Dexamethasone (DEX) was used as a reference. Values are presented as the mean ± SD (standard deviation) of three independent experiments in triplicate. Statistical analyses were carried out by using one-way ANOVA and Tukey post-hoc test with * *p* < 0.05 and *** *p* < 0.001 against the LPS-stimulated cells.

Nitric oxide (NO) is a signalling molecule, the overproduction of which may induce inflammation and affect cancer promotion, while interleukin 6 (IL-6) and tumour necrosis factor-alpha (TNF-α) are inflammatory cytokines that could enhance cancer cells invasion and progression [26]. Thus, we wanted to verify the effect of usnic acid enantiomers on the production of these three pro-inflammatory agents in LPS-stimulated RAW 264.7 macrophages. The results are presented in Figure 4. A significant inhibition of NO synthesis was observed in macrophages pre-treated with usnic acid enantiomers when compared to LPS-stimulated cells (*p* < 0.001). However, the effect was not dose-dependent, and also no significant differences were noted between both usnic acid enantiomers. Only a weak suppression of TNF-α release was noted in LPS-stimulated macrophages pre-treated with usnic acid enantiomers, but the effect was not significant when compared to LPS-stimulated cells, and no dose-dependent activity was noted. In the case of IL-6, only (+)-usnic acid at the dose of 25 µg/mL significantly decreased its release when compared to LPS-stimulated cells (*p* < 0.05).

Jin et al. (2008) indicated a significant inhibitory effect of (+)-usnic acid on NO production and TNF-α release in RAW 264.7 cells, with IC_50_ 12.8 µM and 4.7 µM, respectively [27]. A similar effect was described for (+)-usnic acid at the concentration of 10 µg/mL, with a significant decrease in NO, TNF-α and IL-6, when compared to untreated but LPS-stimulated macrophages [28]. In view of such discrepancies in the results, further studies are certainly needed. 

**Figure 5:**
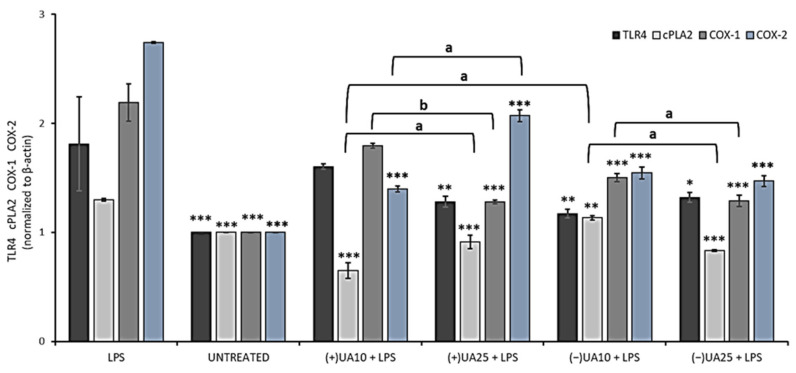
The effect of (+)- and (−)-usnic acid on toll-like receptor 4 (TLR4), cytosolic phospholipase A2 (cPLA2), cyclooxygenase-1 (COX-1) and cyclooxygenase-2 (COX-2) release in LPS-stimulated RAW 264.7 macrophages. RAW cells were treated with 10 (UA10) and 25 (UA25) µg/mL of usnic acid enantiomers and 10 ng/mL of LPS for 24 h. Control cells were not treated with the tested compounds nor LPS (untreated). Densitometric evaluation of the bands TLR4, cPLA2, COX-1, COX-2 from Western blots in relation to beta-actin is shown. Values are presented as the mean ± SD (standard deviation) of three independent experiments in triplicate. Statistical analyses were carried out by using one-way ANOVA and Tukey post-hoc test with * *p* < 0.05, ** *p* < 0.01 and *** *p* < 0.001 against the LPS-stimulated cells. Significant differences (a: *p* < 0.01 and b: *p* < 0.001) between usnic acid enantiomers and doses used in the experiment were marked by upper black line.

In the next step of the current experiment, the influence of usnic acid enantiomers on the synthesis of some proinflammatory proteins was performed by means of Western blot analysis. We focused on the toll-like receptor 4 (TLR4), cytosolic phospholipase A2 (cPLA2) and cyclooxygenases (COX-1, COX-2). The results are presented in Figure 5. TLR4 is an LPS-specific receptor, the activation of which can not only trigger a variety of inflammatory responses [27] but also lead to cancer progression [29]. Both (+)- and (−)-usnic acid at the concentration of 25 µg/mL decreased TLR4 synthesis when compared to LPS-treated macrophages (*p* < 0.01 and *p* < 0.05, respectively), but no significant differences were noted between both enantiomers. Moreover, a similar inhibitory effect (Figure 5) was also observed in macrophages treated with 10 µg/mL of (−)-usnic acid (*p* < 0.01). 

cPLA2 plays an important role in the regulation of the synthesis of arachidonic acid metabolites, which are substrates for COX. We have demonstrated that both usnic acid enantiomers significantly decreased cPLA2 synthesis when compared to the LPS-stimulated macrophages. At 10 µg/mL (+)-usnic acid was a more potent inhibitor of cPLA2 synthesis than its left-handed enantiomer (*p* < 0.01). Moreover, the effect was dose-dependent (*p* < 0.01) for both enantiomers (Figure 5).

COX is an endogenous enzyme existing in two isoforms: COX-1 and COX-2, both of which are involved in the production of prostaglandins and thromboxane from arachidonic acid. The right-handed usnic acid dose-dependently lowered COX-1 protein level (*p* < 0.001), but only at the higher dose the effect differed from the LPS-stimulated macrophages (*p* < 0.001). For (−)-usnic acid, the inhibition was significant at both doses when referred to the LPS-stimulated macrophages (*p* < 0.001). Both usnic acid enantiomers significantly decreased COX-2 protein level when compared to LPS-stimulated macrophages (*p* < 0.001). For (-)-usnic acid, the effect was not dose-dependent, while (+)-usnic acid at a higher dose slightly stimulated COX-2 synthesis. These results are comparable to those obtained by Huang et al. (2014), who showed that (+)-usnic acid at the concentration of 10 µg/mL significantly decreased COX-2 protein level [28]. Our results also indicated the slight pro-inflammatory effect of (+)-usnic acid, as at the higher dose of 25 µg/mL, the compound increased the expression of cPLA2 and COX-2, while for the left-handed enantiomer, such effect was not observed. The difference is very interesting; however, it cannot be explained yet and needs further study. Our results are probably the first to describe the impact of usnic acid enantiomers on TLR4, cPLA and COX-1 regulation, which may complete the so far published information on the anti-inflammatory mechanism of these compounds and their possible role in preventing cancer-related inflammation.

### 2.7. (+)-Usnic Acid Strongly Inhibited Hyaluronidase Activity

Hyaluronidase is an enzyme catalysing the degradation of hyaluronic acid, a key element of connective tissue, hence increasing tissue permeability, which may enable the inflammation process. Thus, the anti-hyaluronidase potential of usnic acid enantiomers was examined by means of a turbidimetric assay, and the results are presented in Table 3. Both enantiomers revealed high and comparable inhibitory effects. The predominant activity of (+)-usnic acid was observed only at the dose of 250 (*p* < 0.05) and 1000 (*p* < 0.001) µg/mL. Moreover, the activity of the right-handed enantiomer was comparable to quercetin, used as a reference drug, except for the dose of 750 µg/mL (*p* < 0.001). 

Anti-hyaluronidase activity of (+)-usnic acid was previously described only once, with 33.14% of inhibition noted at the concentration of 100 µg/mL [30], but the results are difficult to compare due to the different assay conditions used. The results of our study indicate that both usnic acid enantiomers have anti-hyaluronidase potential, which may be another argument for considering these compounds as anti-melanoma agents.

## 3. Materials and Methods

### 3.1. Reagents and Instruments

Dulbecco’s Modified Eagle’s Medium F12 HAM (DMEM/F12), Dulbecco’s Modified Eagle’s Medium with 4500 mg/L glucose (DMEM high glucose), Foetal Bovine Serum (FBS), Trypsin-EDTA solution, Penicillin-Streptomycin solution, Phosphate Buffered Saline (PBS), crystal violet, formaldehyde, Triton X100, DMSO, kojic acid, *L*-tyrosine, *L*-DOPA, mushroom tyrosinase, lipopolysaccharide (LPS), hyaluronidase from bovine testes type I-S, *Streptococcus equi* hyaluronic acid (HA), albumin from bovine serum: fraction V ≥ 98% (A3294), cetyltrimethylammonium bromide (CTAB), quercetin dihydrate, dexamethasone, doxorubicin were purchased in Sigma-Aldrich (Seelze, Germany). Acetate buffer pH 4.5 was purchased from J.T. Baker Chemical Co. (Phillipsburg, NJ, USA). Sodium chloride, sodium hydroxide were purchased from POCH (Gliwice, Poland). Both (+)- and (−)-usnic acid were obtained by isolation from *Cladonia arbuscula* and *C. uncialis*, respectively, as described previously [9,13]. Human malignant melanoma A375 (ATCC CRL-1619) and murine RAW 264.7 macrophages (ATCC TIB-71) were purchased from Sigma-Aldrich, human melanoma HTB140 (ATCC Hs 294T) and WM793 (RRID:CVCL 8787) cell lines were kindly provided by Prof. Marta Michalik from the Department of Cell Biology, Jagiellonian University in Kraków, Poland. Lactate dehydrogenase assay kit (LDH) was obtained from Clontech (Mountain View, CA, USA). Griess Reagent Kit (Promega Corporation (Madison, Winooski, VT, USA), IL-6 and TNF-alpha Human ELISA kits (Bioassay Technology Laboratory, Shanghai, China) were used in the study. M-PER buffer, primary antibodies: anti-COX-1, anti-COX-2, anti-TLR4, anti-cPLA2, and anti-β-actin, secondary antibodies: anti-rabbit IgG-HRP were purchased from Thermo Fisher Scientific (Waltham, MA, USA). Protease inhibitor cocktail set III was from Merck (Darmstadt, Germany). Laemmli buffer, mercaptoethanol, TBS (tris-buffered saline)–1% Tween buffer, Clarity Western ECL Luminol Substrate detection kit were purchased from Bio-Rad (Hercules, CA, USA). Transwell culture plates 6.5 mm, with an 8.0 µm pore polycarbonate membrane, were purchased from Corning Incorporated (New York, NY, USA). Inverted microscope (Leica DMi1 equipped with camera MC120 HD), multi-detection microplate reader (Multi-Detection Microplate Reader Synergy^TM^ HT—BioTek Instruments Inc., Winooski, VT, USA), Chemi Doc Camera with Image Lab software 6.1 (Bio-Rad, Hercules, CA, USA) were used in the study.

### 3.2. Cell Culture Conditions 

Experiments were performed on three melanoma cell lines: HTB140, derived from metastatic site: lymph node, malignant melanoma A375, stage I primary melanoma WM793. For anti-inflammatory assay, murine RAW 264.7 macrophages were used. The cells were cultured in a humidified atmosphere with 5% CO_2_ at 37 °C, in DMEM/F12 (WM793) or DMEM high glucose (A375, HTB-140, RAW 264.7), supplemented with 10% FBS, 100 IU/mL penicillin and 10 µg/mL streptomycin. The tested compounds were diluted in the culture media from freshly made stock solution (10 mg/mL in DMSO) to the working concentrations. 

### 3.3. Viability Assay

The cells were seeded onto 96-well plates (1.5 × 10^4^ cells/well) and cultured for 24 h. Then, the culture medium was replaced with the same medium containing different concentrations of usnic acid enantiomers (0 µg/mL to 100 µg/mL). After 24 h and 48 h of incubation, cell viability was determined by LDH assay, as described previously [31]. Doxorubicin was used as a reference cytostatic drug. All analyses were performed in triplicate; the results are expressed as % of cell viability (mean ± SD) and IC_50_ values (concentration at which the viability is inhibited by 50%).

### 3.4. Isobolographic Analysis

Isobolographic analysis was performed to examine the interactions between (+)- or (−)-usnic acid and doxorubicin. The stock solutions of (+)- or (−)-usnic acid combined with doxorubicin were prepared, at fixed ratio (1:1) combinations, based on the following fractions: 1, 1/2, 1/4, 1/6, 1/8 of the respective IC_50_ values. After 24 h and 48 h of incubation, cell viability was determined by LDH assay. For each mixture, IC_50_ values and their combination index (CI) were determined using the CompuSyn programme, freely available at http://www.combosyn.com (accessed on 1 March 2021) [32]. The combination was considered as synergistic, additive, or antagonistic when the combination index was less than, equal to, or greater than one.

### 3.5. Proliferation Assay

To determine antiproliferative activity, the cells were seeded onto 12-well plates (5 × 10^3^ cells/well) and incubated for 24 h. After 24, 48 and 72 h of incubation with or without usnic acid enantiomers (0–100 µg/mL), cell number was determined using crystal violet assay, as described previously [33]. Briefly, the cells were washed with PBS and fixed with 3.7% formaldehyde. Then, crystal violet solution was added for 10 min, followed by washing with PBS. Crystal violet was extracted from cells using 1.33% citric acid, 1.09% sodium citrate in water/methanol (1:1) solution. The absorbance (A) was measured at 570 nm. The proliferation rate was determined by dividing the A of experimental wells by the A of control wells × 100%. 

### 3.6. Transwell Migration Assay

Cell migration was determined as described previously [33]. Cells were serum-deprived for 24 h and seeded in the upper chamber (1 × 10^4^ cells/well) in serum-free culture medium with (10 µg/mL) or without the tested compounds. The same culture medium was placed in the lower compartment. After 24 h, cells were fixed with 3.7% formaldehyde solution and stained with 0.5% crystal violet solution. The number of migrated cells was counted under an inverted microscope in 10 randomly selected fields of view. Experiments were performed in duplicate, in a blind-folded manner. The results are presented as % inhibition of cell migration referred to the control cells.

### 3.7. Anti-Tyrosinase Assay

Tyrosinase activity assay was evaluated as described previously [34]. The tests were performed on 96-well plates. Briefly, mushroom tyrosinase (100 U/mL), diluted ex tempore in PBS (pH 7.0), was mixed with the tested compounds or kojic acid as a reference drug (0.25 µg/mL to 250 µg/mL) and denoted as SE (sample solution with enzyme). Control samples were prepared by replacing the enzyme with buffer and adding sample solution (S, sample solution without the enzyme). Blank solutions were prepared in the medium without the tested compounds but with (BE) or without (B) the enzyme. After 10 min of incubation, substrate solution was added: 0.4 mg/ml *L*-tyrosine (for monophenolase activity) or 0.2 mg/ml *L*-DOPA (for diphenolase activity). All analyses were performed in triplicate. The absorbance was measured at 492 nm using a microplate reader. Tyrosinase inhibition was calculated as follows:

% inhibition= ([(ABE − AB) − (ASE − AS)]*100)/((ABE − AB))(1)

### 3.8. Determination of NO, IL-6 and TNF-Alpha Release in RAW 264.7 Model 

Prior to the anti-inflammatory experiments, the cells were seeded onto 96 multi-well plates (1.5 × 10^5^ cells/well) and incubated with the tested compounds (0–100 µg/mL) for 24 h. Next, cell viability was tested with the LDH assay. All analyses were performed in triplicate, and the results are expressed as % of cell viability (mean ± SD). For anti-inflammatory assays, RAW 264.7 cells were seeded onto 96 multi-well plates (1.5 × 10^5^ cells/well) and pre-treated with the tested compounds (10 µg/mL and 25 µg/mL) for 1 hour, followed by the addition of 10 ng/mL of LPS to induce inflammation process, according to [35]. Dexamethasone (0.5 µg/mL) was used as a reference drug. The incubation was continued for the next 24 h. Cell culture supernatants were used for further analysis.

Nitric oxide level was determined using Griess Reagent Kit, according to the manufacturer’s protocol. The analysis was performed in triplicates, and the absorbance was measured using a microplate reader. Cytokine (TNF-α, IL-6) release level was performed using Human ELISA kits, according to the manufacturer’s protocol. The analysis was performed in triplicates, and the absorbance was measured using a microplate reader.

### 3.9. Western Blot Analysis

RAW 264.7 cells were seeded onto 6-well plates (1.5 × 10^5^ cells/well) and treated with the tested compounds (10 µg/mL and 25 µg/mL) and 10 ng/mL of LPS to induce the inflammation process. After 24 h of incubation, both the media and the cells were collected by scraping. Cell lysates were prepared using M-PER buffer with protease inhibitor cocktail set III. The protein contents in the lysates were determined with the Bradford reaction. Samples (40 μg) were solubilised in Laemmli buffer with 2% mercaptoethanol and then subjected to 10% SDS-polyacrylamide gel electrophoresis as described previously [36]. Membranes were blocked for 1 hour at room temperature in the presence of casein in TBS–1% Tween buffer and incubated overnight at 4 °C with anti-COX-1, anti-COX-2, anti-TLR4, anti-cPLA2, and anti-β-actin (as endogenous control) primary antibodies, diluted to 1:1000. After incubation, the membranes were washed and incubated with secondary antibodies (anti-rabbit IgG-HRP) for 1 hour at room temperature. The proteins were detected using a Clarity Western ECL Luminol Substrate detection kit. The integrated optical density of the protein bands was quantified using a Chemi Doc Camera with Image Lab software.

### 3.10. Anti-Hyaluronidase Assay

Hyaluronidase inhibition, based on turbidimetric assay (formation of insoluble complexes between non-degraded hyaluronic acid (HA) and cetyltrimethylammonium bromide (CTAB)), was determined as described previously [37]. The test was performed on 96-well microplates. Tested compounds were dissolved in DMSO and analysed at final concentrations from 10 μg/mL to 1000 μg/mL. Quercetin was used as a reference drug. Briefly, the examined substances were mixed with incubation buffer, enzyme, and acetate buffer (pH 4.5) and preincubated at 37 °C for 10 min. Then, HA was added to the mixtures and incubation at 37 °C was continued for 45 min. Next, CTAB solution was added to precipitate the undigested HA. The absorbance in the presence of enzyme and substrate (control I) and in the absence of enzyme (control II) was measured at 600 nm. Product control solution, with HA replaced by buffer, was prepared to diminish sample background. The absorbance of the medium was performed as a blind control of the experiment. All experiments were performed in triplicate. The percentage of inhibition was calculated as follows:

% inhibition = {[As − (APc − AB)] − AI }/{[AII − (APc − AB)] − AI} × 100(2)

AI—absorbance of enzyme + substrate (control I);

AII—absorbance in the absence of enzyme (control II); 

AS—absorbance of sample solution; 

APc—absorbance of the product control solution;

AB—absorbance of a blank control of experiment;

The half-maximal inhibitory concentration value IC_50_ was also determined.

### 3.11. Statistical Analysis

The differences between the obtained results were tested using one-way Analysis of Variance (ANOVA) with Tukey-Kramer Multiple Comparisons Post-hoc Test or Bonferroni post-hoc test for anti-hyaluronidase assay. Statistical analysis was done using Statistica v.13 (Statsoft, Tulsa, OK, USA).

## 4. Conclusions

Both tested usnic acid enantiomers revealed significant impact on human melanoma cells functions, but also on inflammation, the symptom often co-existing with cancer. Among the tested enantiomers, (+)-usnic acid seems to reveal more potent cytotoxic and cytostatic properties, with strong inhibition of cells migration. The anti-inflammatory effect of the right-handed usnic acid is also promising, with a varied influence on a panel of pro-inflammatory mediators. These features make (+)-usnic acid a good candidate for an anti-melanoma agent; however, further studies should be continued to better address mechanistic aspects of its action. Moreover, more in-depth studies on the interactions of usnic acid enantiomers with cytostatic drugs are needed regarding the interesting results obtained in the study. Our results also expand the knowledge on usnic acid enantiomers impact on melanoma cells in vitro. The obtained results may have future implications for the development of new strategies for combating melanoma, with the heterogeneity of tumours as the main target. 

## Figures and Tables

**Table 1:** Concentrations of (+)- and (−)-usnic acid (UA) and doxorubicin (DOX) alone or in combinations, that induced 50% decrease in cell viability after 24 h and 48 h of treatment.

Treatment	Incubation	IC_50_ (µg/mL)		
		HTB140	A375	WM793
(+)-UA	24 h	16.99	15.10	45.68
	48 h	14.72	11.84	30.05
(−)-UA	24 h	26.24	29.90	87.95
	48 h	20.62	22.14	52.09
DOX	24 h	3.77	1.59	>100 *
	48 h	2.01	0.55	>100 *
(+)UA + DOX	24 h	12.74	11.80	73.45
	48 h	10.92	6.08	41.61
(−)UA + DOX	24 h	12.56	14.99	33.19
	48 h	9.96	8.08	26.15

* IC_50_ for doxorubicin for WM793 cell line was not possible to calculate in the tested concentration range; thus, for the isobolographic analysis, the IC_50_ value of the appropriate usnic acid enantiomer was also used as the data for doxorubicin.

**Table 2:** Isobolographic analysis of the combinations of (+)- or (−)-usnic acid (UA) with doxorubicin (DOX), performed as combination index (CI).

Treatment		CI after 24 h			CI after 48 h		
		HTB140	A375	WM793	HTB140	A375	WM793
MIX 1 IC_50_	(+)UA + DOX	3.73 ⬤	1.30 ⬤	0.75 ⬤	2.67 ⬤	1.11 ⬤	0.36 ⬤
	(−)UA + DOX	0.54 ⬤	0.70 ⬤	0.003 ⬤	0.24 ⬤	0.52 ⬤	0.05 ⬤
MIX 2 1/2 IC_50_	(+)UA + DOX	2.54 ⬤	0.84 ⬤	0.76 ⬤	2.04 ⬤	0.75 ⬤	0.60 ⬤
	(−)UA + DOX	0.88 ⬤	0.56 ⬤	0.07 ⬤	1.01 ⬤	0.69 ⬤	0.21 ⬤
MIX 3 1/4 IC_50_	(+)UA + DOX	1.78 ⬤	0.52 ⬤	1.06 ⬤	1.44 ⬤	0.57 ⬤	0.82 ⬤
	(−)UA + DOX	0.87 ⬤	0.46 ⬤	0.15 ⬤	1.04 ⬤	0.64 ⬤	0.40 ⬤
MIX 4 1/6 IC_50_	(+)UA + DOX	1.42 ⬤	0.42 ⬤	1.37 ⬤	1.09 ⬤	0.45 ⬤	0.87 ⬤
	(−)UA + DOX	0.88 ⬤	0.45 ⬤	0.24 ⬤	1.25 ⬤	0.54 ⬤	0.52 ⬤
MIX 5 1/8 IC_50_	(+)UA + DOX	1.39 ⬤	0.44 ⬤	1.84 ⬤	1.00 ⬤	0.43 ⬤	1.37 ⬤
	(−)UA + DOX	1.06 ⬤	0.57 ⬤	0.47 ⬤	1.28 ⬤	0.52 ⬤	0.83 ⬤

Mixes were prepared at fixed ratio (1:1), based on the following fractions: 1 (MIX 1), 1/2 (MIX 2), 1/4 (MIX 3), 1/6 (MIX 4), 1/8 (MIX 5) of the respective IC_50_ values; CI < 1 synergism (⬤); CI = 1 additive (⬤); CI > 1 antagonism (⬤).

**Table 3:** Anti-hyaluronidase activity of (+)- and (−)-usnic acid.

Concentration (µg/mL)	% Hyaluronidase Inhibition (Mean ± SD)		
	(+)-Usnic Acid	(−)-Usnic Acid	Quercetin
50	0.00 ± 0.00	0.00 ± 1.82	1.23 ± 0.99
100	0.62 ± 1.07 ^a^	3.70 ± 0.42	4.39 ± 0.88
250	13.69 ± 4.49 *	23.33 ± 3.33	18.10 ± 1.01
500	37.81 ± 7.51	40.80 ± 3.59	38.84 ± 1.33
750	44.26 ± 2.84 ^a^	42.29 ± 4.56 ^a^	87.27 ± 1.16
1000	88.52 ± 1.64 ***	72.33 ± 9.31 ^a^	91.11 ± 1.50
IC_50_	644.45	676.27	517.33

Hyaluronidase inhibition was based on turbidimetric assay. All assays were performed in triplicate. Statistical analyses were carried out by using one-way ANOVA and Tukey post-hoc test. ^a^ difference statistically significant vs control (*p* < 0.001); *difference between the activity of both enantiomers (* *p* < 0.05, *** *p* < 0.001).

## Data Availability

Data is contained within the article.
